# EZH2 is crucial for both differentiation of regulatory T cells and T effector cell expansion

**DOI:** 10.1038/srep10643

**Published:** 2015-06-19

**Authors:** Xiang-Ping Yang, Kan Jiang, Kiyoshi Hirahara, Golnaz Vahedi, Behdad Afzali, Giuseppe Sciume, Michael Bonelli, Hong-Wei Sun, Dragana Jankovic, Yuka Kanno, Vittorio Sartorelli, John J. O’Shea, Arian Laurence

**Affiliations:** 1 Department of Immunology, School of Basic Medicine, Tongji Medical College, Huazhong University of Science and Technology, Wuhan, 430030, China; 2 Lymphocyte Cell Biology Section, Molecular Immunology and Inflammation Branch National Institutes of Arthritis, and Musculoskeletal and Skin Diseases National Institutes of Health, Bethesda, MD 20892, USA; 3 MRC Centre for Transplantation, King’s College London, UK; 4 Biodata Mining and Discovery Section, National Institute of Arthritis and Musculoskeletal and Skin Diseases, National Institutes of Health, Bethesda, MD 20892-1930, USA; 5 Immunobiology Section, Laboratory of Parasitic Diseases, National Institute of Allergy and Infectious Diseases; 6 Laboratory of Muscle Stem Cells and Gene Regulation, National Institute of Arthritis and Musculoskeletal and Skin Diseases, National Institutes of Health, Bethesda, MD 20892-1930, USA; 7 Northern Centre for cancer care, Freeman hospital, Newcastle upon tyne, UK; 8 NDM Experimental medicine, John Radcliffe hospital, Oxford, UK

## Abstract

The roles of EZH2 in various subsets of CD4^+^ T cells are controversial and its mechanisms of action are incompletely understood. FOXP3-positive Treg cells are a critical helper T cell subset, and dysregulation of Treg generation or function results in systemic autoimmunity. FOXP3 associates with EZH2 to mediate gene repression and suppressive function. Herein, we demonstrate that deletion of *Ezh2* in CD4 T cells resulted in reduced numbers of Treg cells *in vivo* and differentiation *in vitro* and an increased proportion of memory CD4 T cells in part due to exaggerated production of effector cytokines. Furthermore, we found that both *Ezh2-*deficient Treg cells and T effector cells were functionally impaired *in vivo*: Tregs failed to constrain autoimmune colitis and T effector cells neither provided a protective response to *T. gondii* infection nor mediated autoimmune colitis. The dichotomous function of EZH2 in regulating differentiation and senescence in effector and regulatory T cells helps to explain the apparent existing contradictions in literature.

Upon encounter with cognate antigen, naïve CD4^+^ T cells differentiate into a variety of distinct subsets including: T helper1 (Th1), Th2, and Th17 that are characterized by the secretion of selective cytokines. Each subset is able to orchestrate a particular immune response and in this way control a wide range of invasive pathogens^1^,^2^. Opposing these effector cell lineages are T regulatory (Treg) cells, characterized by the expression of the transcription factor FOXP3. Treg cells can be generated in the thymus (tTreg cells) or induced in the periphery (pTreg) or *in vitro* (iTreg) from naïve T cells activated in the presence of transforming growth factor (TGF)-β and interleukin (IL)-2. Given the central roles of CD4 T cells in instructing appropriate host immune responses, the process of CD4 differentiation is tightly regulated by a network of transcriptional factors and epigenetic changes^2^,^3^.

The contribution of epigenetic modifications to Th cell differentiation has attracted recent interest^3^,^4^. One relevant factor is methylation of the *Foxp3* locus^5^, but in addition, post-translational modifications of histones represent another factor that can alter the chromatin accessibility. Among the multiple histone modifications, trimethylation of histone 3 lysine 4 (H3K4m3) is often associated with active transcription whereas trimethylation of histone 3 lysine 27 (H3K27m3) is a transcriptional suppression mark^6^. The generation of H3K27m3 is mediated by Polycomb-Repressive Complex 2 (PRC2), initially identified as negative regulators of the homeotic genes, which are essential for proper segmentation in *Drosophil*a. The mammalian PRC2 contains SUZ12, EED, RbAp48, and EZH2, the catalytic subunit of PRC2 for the generation of H3K27m3^7^,^8^. Deletion of EZH2 is embryonically lethal and mouse genetic studies with conditional deletion of EZH2 in multiple tissues revealed that critical roles of EZH2-mediated H3K27m3 in regulating cell proliferation, cell differentiation, stem cell identity and pluripotency^9^. Aberrant activation of EZH2 has been associated with development of various malignances^10^,^11^,^12^.

Although it has been intensively studied, the function of EZH2 in T cell differentiation remains controversial. Jacob *et al.* reported that EZH2 binds to IFN-γ promoter in differentiating Th1 but not Th2 cells and the authors concluded that EZH2 plays an unconventional positive role in mediating both Th1 and Th2 differentiation^13^. In line with this, the group of Zhang found that EZH2 is required for both *in vivo* and *in vitro* Th1 generation and Th1-mediated graft-versus-host disease by multiple mechanisms: binding to *Tbx21* promoter and inducing *Tbx21* expression, and suppressing proteasome-mediated T-bet degradation^14^,^15^. In contrast, other groups showed that deletion of EZH2 leads to increased Th1 and Th2 differentiation, suggesting that EZH2 suppress both Th1 and Th2 differentiation^16^,^17^. Several groups have noted a survival difference between wild type and *Ezh2*-deficient T cells, yet this again remains controversial as the mechanism by which this happens is not consistent. He and colleagues identified a defect in Bim expression whereas the work by Zhang *et al.* identified a defect in caspase signaling^14^,^17^. Recent work has shown that when activated, FOXP3 co-localizes with EZH2, suggesting that the latter may be required for the repression of inflammatory gene expression by FOXP3^18^ and in the absence of EZH2 *in vitro,* iTreg differentiation has been shown to be impaired^17^. Furthermore, mice that lack EZH2 in only FOXP3-expressing cells develop autoimmune disease^19^.

Herein, we investigated the impact of EZH2 on Treg cell function. We found that absence EZH2 resulted in diminution in Treg cell numbers with a concomitant expansion of memory T cells. Absence of EZH2 also interfered with Treg cell function and impaired expression of FOXP3 as a consequence of the overproduction of effector cytokines. However, effector T cell function was also impaired; these cells were unable to provide protective responses in *T. gondii* infection and did not mediate disease in a model of autoimmune colitis. Finally, we found that absence of EZH2 has a profound role in regulation of cellular senescence. Thus, the absence of autoimmunity in the face of defective Treg cell function in mice lacking EZH2 in CD4 cells is explained by the concomitant defects in effector T cells. These data help to explain some of the apparent existing contradictions in the literature.

## Results

### *Ezh2*-deficient mice have fewer Treg cells and more memory T cells

**Figure 1:**
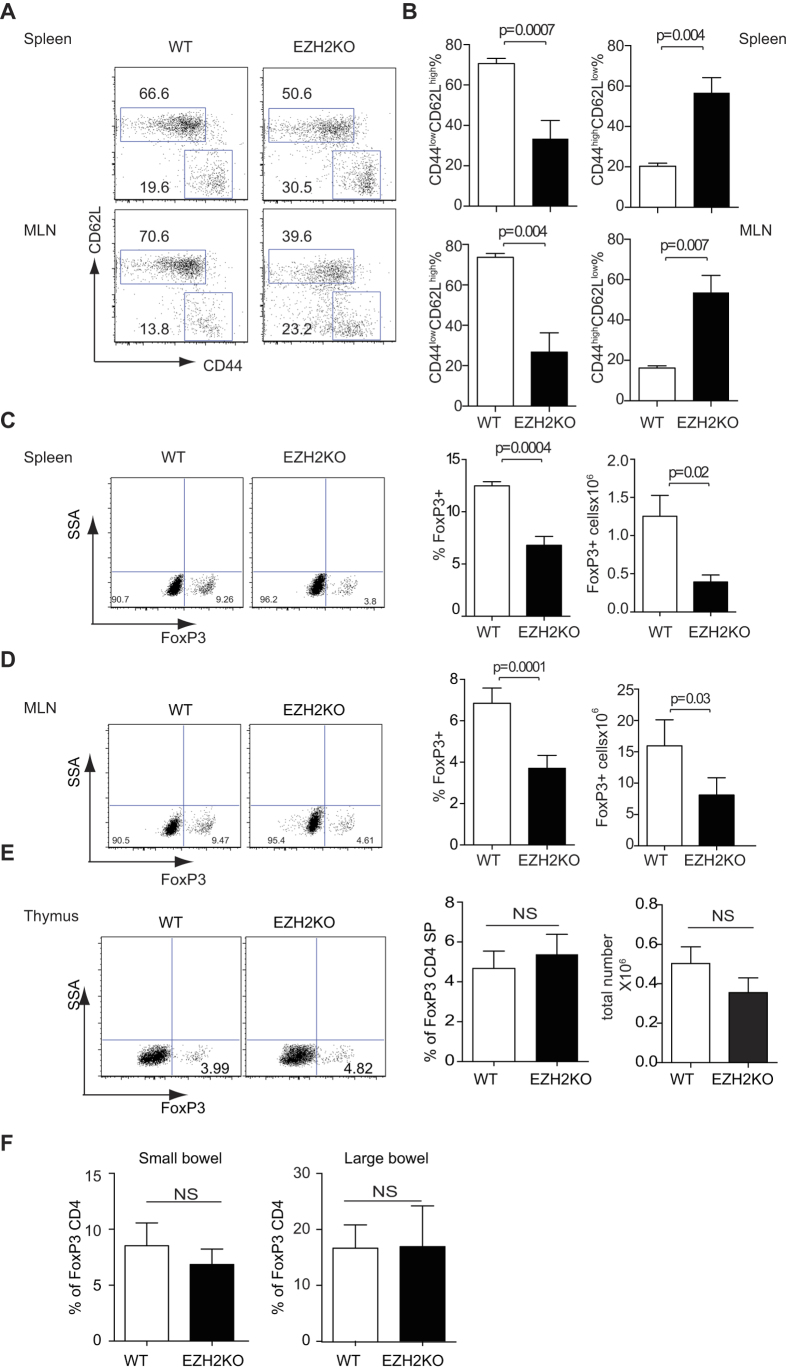
*Ezh2*-deficient mice have fewer naïve T cells in their periphery. (**A**) CD44 and CD62L expression of spleen and mesenteric CD4^+^ T cells from control and *Ezh2*-deficient mice. Data are representative of six independent experiments. (**B**) Cumulative histogram showing percentage of CD4^+^CD44^low^CD62L^high^ and CD4^+^CD44^high^CD62L^low^ in the spleen and mesenteric lymphocytes of control and *Ezh2*-deficient mice. ^*^P < 0.05 and ^**^P < 0.01 (unpaired *t*-test) (n* *= 6). FOXP3 expression in CD4^+^ T cells as measured by intracellular staining and flow cytometery of lymphocytes from the spleen (**C**) mesenteric lymph nodes (**D**), thymus (**E**), small and large bowel (**F**). Representative dot plots demonstrating FOXP3 expression are shown on the left, histograms showing pooled data depicting the average proportion of FOXP3^+^ cells together with total numbers of FOXP3^+^ cells are shown on the right, error bars denote the s.e.m. ^*^P < 0.05 and ^**^P < 0.01 (unpaired *t*-test) (n* *= 3) (**C**–**E**). (**F**) Histograms depict average percentage of FOXP3^+^ T cells as a proportion of the total CD4^+^ population in the small and large bowel, error bars denote s.e.m. ns denotes no significance (n* *= 3).

The function of EZH2 in CD4 differentiation is controversial, with both positive and negative roles of EZH2 in regulation of Th1 and Th2 being reported^13^,^20^,^21^. Deletion of EZH2 in the bone marrow resulted in blockade of thymocyte development in the double negative compartment prior to the checkpoints of positive and negative selection^22^. To investigate the function of EZH2 in T cells we bred *Ezh2*^*fl/fl*^ mice with *CD4-Cre* transgenic mice. The resulting *CD4-Cre; Ezh2*^*fl/fl*^ animals are viable with no obvious phenotype up to nine months of age, in keeping with previous reports^14^,^16^. Separating naïve and activated T helper cells on the basis of CD44 and CD62L expression, we found that the percentage and numbers of activated T helper cells were significantly increased, while both the frequency and numbers of naïve Th cells were significantly reduced in the spleens of the *CD4-Cre; Ezh2*^*fl/fl*^ mice (Fig. 1A,B). The observed spontaneous activation of CD4 T cells in the *Ezh2*-deficient mice was more evident with age compared with control animals (**Supplementary Fig. 1A**).

Loss of naïve CD4^+^ T cells can be associated with defects in either Treg function or numbers. We next sought to investigate the possibility that dysregulation of this subset explained the loss of naïve T cells. To this end, we measured the proportion of CD4^+^ T cells that expressed FOXP3 and found that in the spleen and mesenteric lymph nodes of *CD4-Cre; Ezh2*^*fl/fl*^ mice, both the percentage and numbers of FOXP3^+^ cells were significantly reduced (Fig. 1C,D). However, there was no significant difference in the proportions and absolute number of FOXP3-expressing tTreg in *CD4-Cre; Ezh2*^*fl/fl*^ mice and WT mice (Fig. 1E). Similarly, the proportions of FOXP3-expressing pTreg in both small and large intestine were also similar between WT mice and *Ezh2*-deficient mice (Fig. 1F). Consistent with this finding the colons of aged *Ezh2*-deficient mice had no evidence of colitis (**Supplementary Fig. 1B**). Taken together, ablation of EZH2 in the T cell compartment led to the presence of more activated T cells and a reduced percentage of FOXP3^+^ CD4^+^ cells in the secondary lymphoid organs. Yet this did not translate into the development of tissue inflammation and damage.

### Neutralizing IFN-γ and IL-4 reverses impaired *FoxP3* expression in *Ezh2*-deficient T cells

**Figure 2:**
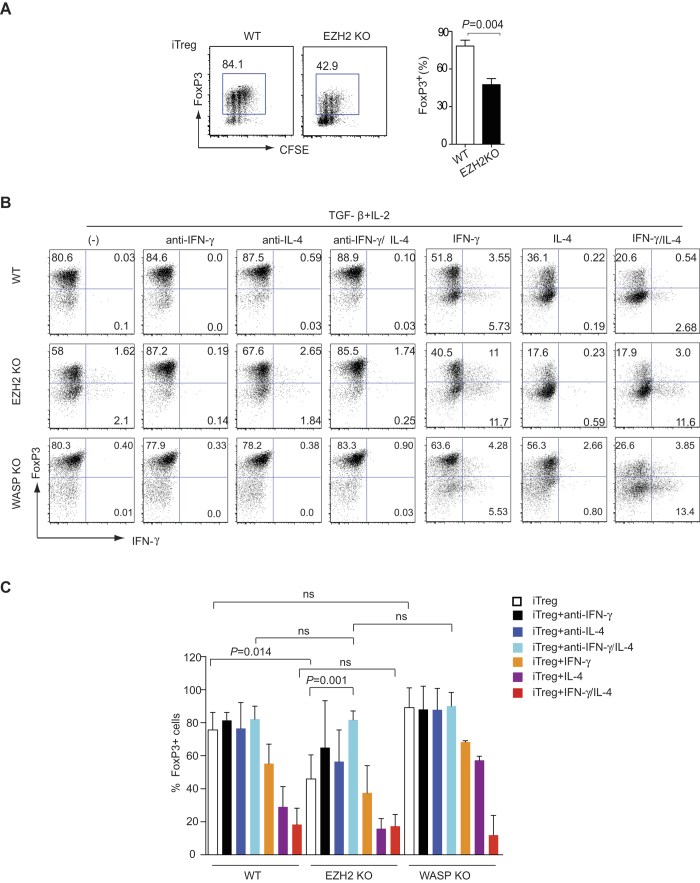
Addition or inhibition of IFN-γ and IL-4 resolves the impaired iTreg differentiation seen in *Ezh2*-deficient T cells. (**A**) CFSE staining and intracellular staining of FOXP3 in activated CD4^+^ T cells obtained from naive CD4^+^ T cells from control or *Ezh2*-deficient mice (EZH2-KO), stimulated for 3 days under iTreg conditions. The histograms represent pooled data from four independent experiments, error bars denote s.e.m. *P* values were calculated using an unpaired *t*-test. (B-C) Naïve CD4^+^ T cells were isolated from control (WT), *Ezh2*-deficient (EZH2-KO) or *WASP*-deficient (WASP-KO) mice and stimulated in the presence of IL-2 and TGF-β alone, or with anti-IFN-γ, anti-IL-4, both anti-IFN-γ and anti-IL-4, IFN-γ, IL-4 or both IFN-γ and IL-4 for 3 days, followed by intracellular staining of FOXP3 and IFN-γ. Dot plots are reperesentative of three independent experiments (**B**) Histograms denote mean values (n* *= 3), error bars denote s.e.m. (**C**). *P* values were calculated using an unpaired *t*-test..

We next sought to determine potential mechanisms underlying the partial reduction in FOXP3^+^ T cells in *CD4-Cre; Ezh2*^*fl/fl*^ mice. To dissect a potential mechanism, we stimulated control and *Ezh2*-deficient naïve T cells under iTreg conditions and measured cell proliferation by dilution of CFSE and induction of FOXP3 expression. After three days we found no differences in activation and proliferation of *Ezh2*-deficient cells as determined by their ability to dilute CFSE. However, we did find a significant reduction in the expression of FOXP3 (Fig. 2A).

One potential explanation for the failure to induce FOXP3 expression was defective T cell receptor signaling, as EZH2 is known to play a role in actin polymerization and formation of the immune synapse. To interrogate the possible involvement of actin polymerization in the regulation of Treg differentiation, we also included *WASP*-deficient T cells as control. WASP, like EZH2, is essential in mediating actin polymerization through activation of Arp2/3 complex in T cells and patients with mutations of WASP are immunodeficient^23^. However, we did not see a significant difference in the ability of *WASP*-deficient T cells to express FOXP3 when stimulated under iTreg conditions (Fig. 2B – left most panels).

We next sought to identify other mechanisms underlying the failure of *Ezh2*-deficient T cells to induce FOXP3. STAT1 activation downstream of IFN-γ and STAT6 activation downstream of IL-4 have both been implicated in inhibiting *FoxP3* expression^24^. We wondered whether the impaired expression of FOXP3 seen in *Ezh2*-deficient iTreg cultures was secondary to aberrant expression of effector cytokines that could feedback to inhibit *FOXP3* expression. To address these questions, control and *Ezh2*-deficient naïve CD4 cells were stimulated in the presence of TGF-β and IL-2, with or without neutralizing anti-cytokine antibodies. We found that neutralizing IFN-γ and IL-4 individually or together had little effect on FOXP3 expression in WT cells and *WASP*-deficient cells. By contrast, neutralizing IFN-γ or IL-4 alone lead to a significant increase in FOXP3 expression in *Ezh2*-deficient cells (*P *= 0.032, *P *= 0.046 respectively) and the combination of anti-IFN-γ and anti-IL-4 completely reversed the deficit in FOXP3 expression (Fig. 2B left four panels, 2C).

Next, we asked if addition of exogenous IFN-γ and IL-4 would be sufficient to recapitulate the impairment of *Foxp3* expression. We found that IL-4 had a stronger inhibitory effect on FOXP3 expression than IFN-γ (Fig. 2B right panels, 2C) and the addition of both cytokines had an addictive effect resulting in only 20% of control cells expressing FOXP3. In the presence of exogenous IFN-γ and IL-4, FOXP3 expression in both control and *Ezh2*-deficient T cells was similar (Fig. 2C right columns). These data indicate that defective induction of FOXP3 in *Ezh2*-deficient cells is attributable to aberrant production of IFN-γ and IL-4 and not due to other cell-intrinsic mechanisms.

### *Ezh2*-deficient Treg cells fail to protect against experimental colitis

**Figure 3:**
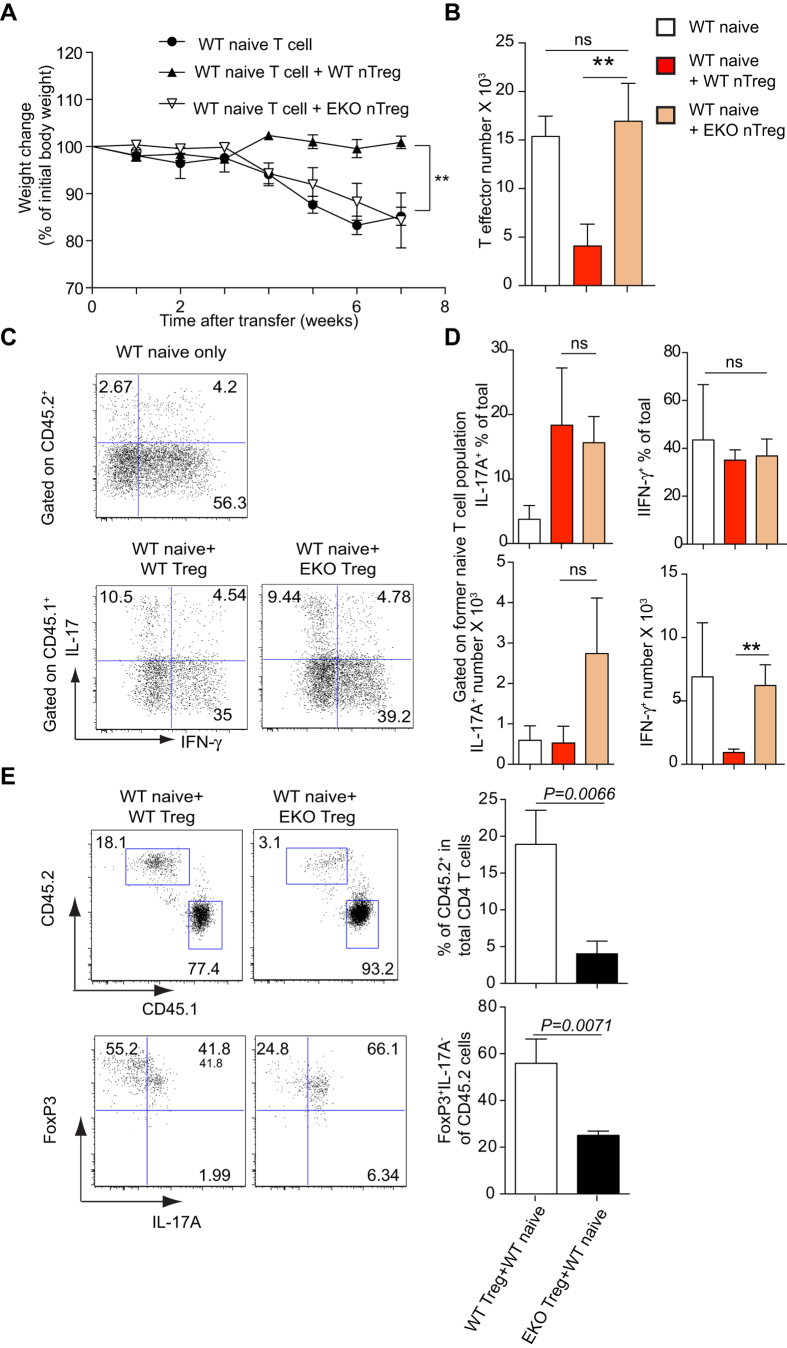
*Ezh2*-deficient Treg cells fail to prevent wild type naïve Th cells driven transfer colitis. Sorted 4 × 10^5^ CD4^+^CD45RB^hi^CD25^−^ cells from control CD45.2 mice were injected intravenously into *Rag2*^−/−^ mice alone (n* *= 5) or sorted 4 × 10^5^ CD4^+^CD45RB^hi^CD25^−^ cells from CD45.1 congenic mice together with sorted 1 × 10^5^ CD4^+^GFP^+^CD25^+^NRP1^+^ from either *Ezh2*^*fl/fl*^*;Foxp3-GFP* mice (WT nTreg) or *CD4-Cre*;*Ezh2*^*fl/fl*^*;Foxp3-GFP* mice (EKO nTreg) were co-injected intravenously into *Rag2*^−/−^ mice (n* *= 3). Mice were monitored for evidence of colitis. (**A**) Weight loss (percentage of initial weight at day 0) was calculated for each mouse over 7 weeks. Data show mean (±s.e.m) weight change for each group (^**^P < 0.01). (**B**) Histogram denotes the mean total numbers of transferred T effector cells (gated on CD45.2 from the injection of WT naïve group; gated on CD45.1 from the co-injection groups of both naïve and Treg cells) that were recovered from the lamina propria after 7 weeks. ^**^P < 0.01 (unpaired *t*-test). (**C**) Intracellular staining of IL-17 and IFN-γ of CD4^+^ T effector cells recovered from lamina propria after 7 weeks. (**D**) Histograms denote the mean percentage (±s.e.m) of transferred CD4^+^ T effector cells that were either IFN-γ^+^ or IL-17^+^ in the lamina propria of *Rag2*^−/−^ mice after 7 weeks (n* *= 3) (upper panels); the mean (±s.e.m) absolute cell numbers of IL-17^+^CD4^+^ and IFN-γ^+^CD4^+^ in the lamina propria of *Rag2*^−/−^ mice were enumerated (lower panel) (n* *= 3) ^**^P < 0.01 (unpaired *t*-test). (**E**) The percentages of CD45.2^+^ (Treg fraction) and CD45.1^+^ (T effector fraction) in CD4^+^ T cell compartment in the lamina propria of *Rag2*^−/−^ mice after 7 weeks were determined by flow cytometry (left upper panels). Histograms depict the mean percentage of CD45.2^+^ from the total CD4 T^+^ cell population (right upper panel). Representative experiments depict FOXP3 and IL-17A expression as measured by intracellular staining in the CD45.2^+^ compartment (left lower panels). Histograms depict the mean percentage of FOXP3^+^IL-17A^−^ in CD45.2^+^ T cells (right lower panel). Error bars denote the s.e.m. Significance was determined using the unpaired *t*-test (n* *= 3).

Having shown that FOXP3 can be induced in *Ezh2*-deficient iTreg cells when effector cytokines were blocked, we next investigated the ability of these cells to suppress inflammation *in vivo* using an established T cell transfer model. Specifically, we adoptively transferred naïve CD4^+^ T cells from either WT or *Ezh2-*deficient mice (CD45.2), or sorted CD4^+^FOXP3^+^CD25^+^Nrp1^+^ tTreg cells from control mice or *Ezh2-*deficient mice (CD45.2) together with WT naïve CD4 T cells from (CD45.1) congenic mice into *Rag2*^*−/−*^ mice. As expected, we observed that transfer of WT naïve cells resulted in colitis and weight loss, which was ameliorated by the simultaneous transfer of WT Treg cells. By contrast, mice that received WT naïve CD4^+^ T cells together with *Ezh2-*deficient Treg cells developed colitis and lost weight (Fig. 3A), suggesting that *Ezh2*-deficient Tregs were functionally defective.

Next, we determined the numbers of CD4^+^ T effector cells from the gut (CD45.2^+^ cells from WT and *Ezh2-*deficient naïve groups, CD45.1^+^ cells from the mice injected with both Treg and naïve T cells). We found the T effector numbers correlated with disease severity: the numbers of T effector cells from mice injected with either control naïve T cells or control naïve T cells together with *Ezh2*-deficient Treg cells were significantly greater than the numbers of T effector cells from mice simultaneously injected with control naïve T cells and control Treg cells (Fig. 3B).

Both Th1 and Th17 cells have been implicated in the pathogenesis of T cell-mediated colitis^25^. To determine whether EZH2 plays a critical role in the ability of Tregs to suppress these lineages, we determined IL-17^+^ and IFN-γ^+^ T effector cells within inflamed lamina propria following adoptive transfer. The presence of WT, but not *Ezh2*-deficient, Tregs resulted in a significant reduction in the total number of IFN-γ^+^ T cells isolated from lamina propria at the end of the experiment (Fig. 3C,D), demonstrating impaired ability of *Ezh2*-deficient Tregs to suppress Th1 inflammation *in vivo*.

To test if deficiency of EZH2 in Treg cells could alter the expansion of Treg and T effector cells *in vivo*, we also measured the percentages of Treg and T effector cells in the CD4 compartment at the end of colitis induction. After 7 weeks, *Ezh2*-deficient Treg cells accounted for only 4.03% (± 0.99 SEM) of the CD4^+^ T cells, whereas the percentage of wild type Treg cells was significantly higher (18.9%, ± 2.68 SEM) (Fig. 3E), suggesting that EZH2 is required for Treg cell expansion *in vivo* during inflammation. Alternatively, EZH2 may be required to maintain the stability of Treg cells in a cell-intrinsic manner. To test this, we determined the proportions of FOXP3 and IL-17A expression in the transferred Treg cells. In the absence of EZH2, there was a significantly lower percentage of FOXP3^+^IL-17A^−^ cells in the gut, which maintained this phenotype (25.1%, ± 1.04 SEM) compared to their wild type counterpart (55.9%, ± 5.98 SEM) (Fig. 3E), suggesting that EZH2 is required for the maintenance of Treg stability in inflammatory conditions. In line with this, when isolated CD4^+^CD25^+^Nrp1^+^ tTreg cells from WT and *Ezh2*-deficient spleen and MLN were stimulated with IL-12, *Ezh2*-deficient Treg cells lost FOXP3 expression to a greater extent, becoming IFN-γ-producing cells (**Supplementary Fig 2**).

### EZH2 represses effector differentiation *in vitro*, but is required for fully functional effector cells *in vivo*

Our data indicated that *Ezh2*-deficient mice had reduced numbers of FOXP3^+^ CD4 cells *in vivo*, and reduced induction of FOXP3 *in vitro*. This was associated with impaired Treg cell function. Mice with similar defects typically develop autoimmune disease^26^,^27^ and although we saw an increased proportion of activated T cells in *Ezh2*-deficient mice compared with littermate controls, we saw little evidence of the development of spontaneous autoimmune disease. For example, aged *Ezh2*-deficient mice had no evidence of colitis (**Supplementary Fig. 1B**). We postulated that an explanation for these data were co-existing abnormalities in effector T cells.

**Figure 4:**
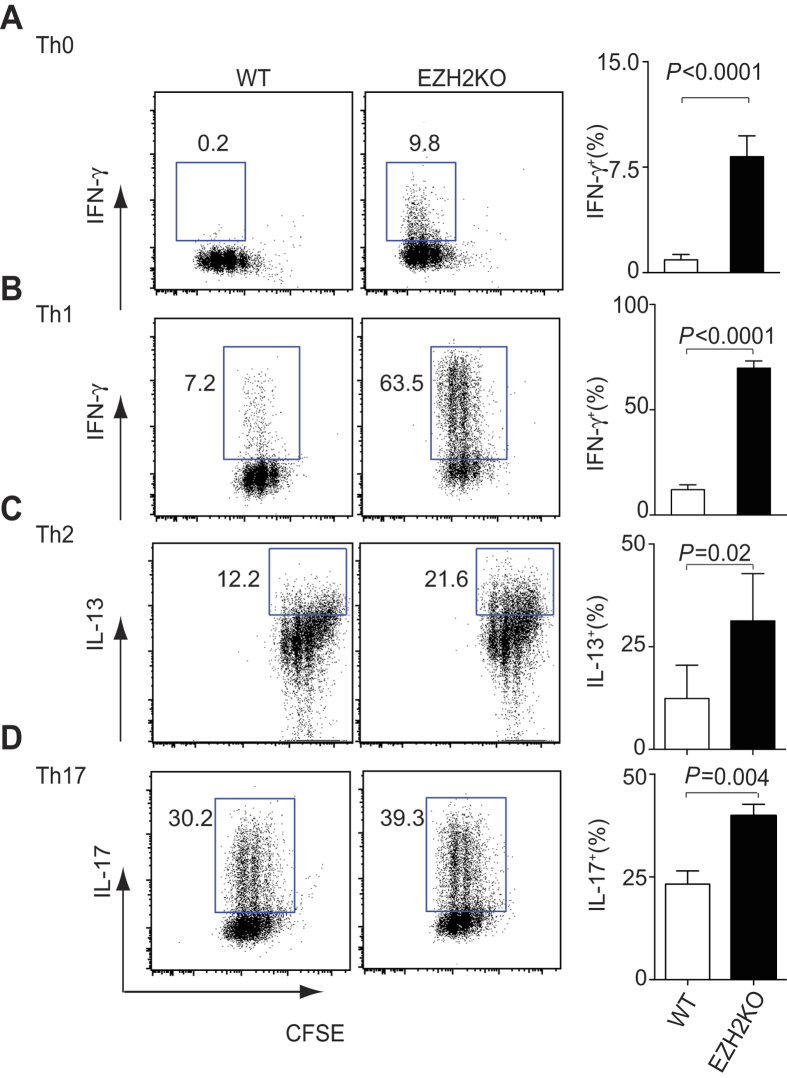
EZH2 suppresses Th1, Th2 and Th17 differentiation. (**A**–**D**) CFSE staining and intracellular staining of IFN-γ (**A**,**B**), IL-13 (**C**), IL-17 (**D**) in naive CD4^+^ T cells from control (WT) or *Ezh2*-deficient (EZH2-KO) mice, stimulated for 3 days under either Th0 conditions (Medium alone) (**A**), Th1 conditions (**B**), Th2 conditions (**C**) or Th17 conditions (**D**). The histograms represent mean percentage of cytokine expressing T cells, error bars denote the s.e.m from four independent experiments. *P* values were calculated using a paired *t*-test.

We therefore tested the ability of *Ezh2*-deficient T cells to express effector cytokines *in vitro*. Naïve CD4^+^ T cells were stimulated in the presence of media alone or under Th1, Th2 or Th17 polarizing conditions. In the presence of neutral or Th1 polarizing conditions, *Ezh2*-deficient CD4^+^ T cells produced significantly more IFN-γ compared with control T cells (Fig. 4A,B). Similarly, *Ezh2*-deficient CD4^+^ T cells produced significantly higher amounts of IL-13 and IL-17 in Th2 or Th17 conditions respectively, compared with control T cells (Fig. 4C,D), although the differences were not as marked as under Th1 conditions. Collectively, these results demonstrated that during differentiation, EZH2 suppresses lineage signature cytokines. Given that the effector program was exaggerated in *Ezh2*-deficient cells, we expected that the cells would be more aggressive in mediating host defense and autoimmunity.

### Mice harboring *Ezh2*-deficient T cells succumb to *T. gondii* infection

**Figure 5:**
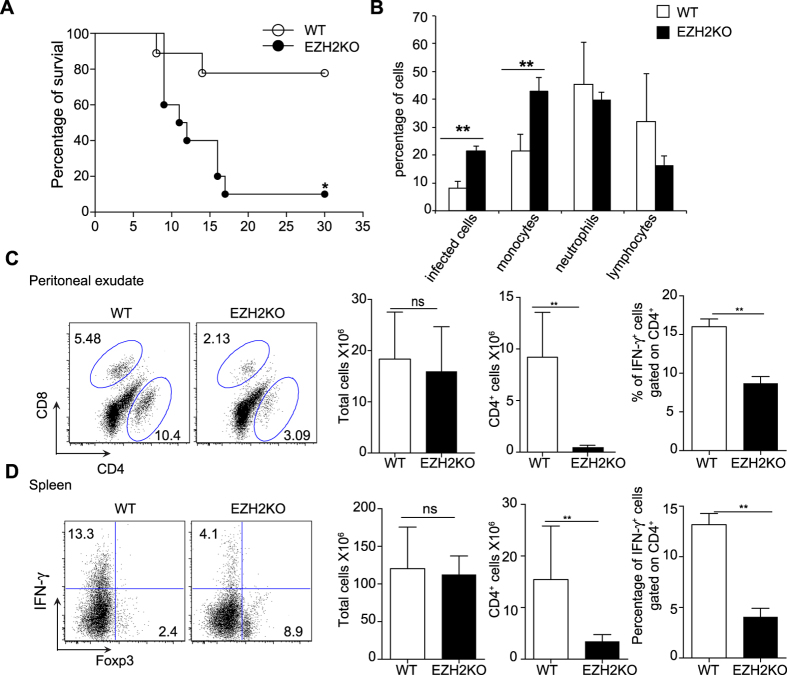
*Toxoplasma gondii* infection of control and *Ezh2*-deficient mice. Control mice (WT) (n* *= 5) and *Ezh2*-deficient mice (EZH2KO) (n* *= 5) were intraperitoneally infected with *Toxoplasma gondii* cysts. (**A**) Survival curve of infected control mice and *Ezh2*-deficient mice post infection. Data represent a cumulative of three independent experiments. (**B**) Cell composition of PEC on day 7 post infection; bars indicate the mean (+/− SEM) percentage of indicated cell type in PEC from control and *Ezh2*-deficient mice mice (n* *= 5). (**C**) CD4 and CD8 staining of peritoneal exudate from control and *Ezh2*-deficient mice (left panel); histogram represents the mean total cell numbers, mean CD4 cell numbers and mean percentage of IFN-γ-producing CD4^+^ T cells in the control and *Ezh2*-deficient mice, error bars denote the s.e.m (n* *= 3). Data are representative of three independent experiments ^*^P < 0.05 and ^**^P < 0.01 (unpaired *t*-test). (**D**) Intracellular staining of IFN-γ and FOXP3 in the splenocytes of control and *Ezh2*-deficient mice 14 days after infection (left panel); histograms (right panels) show the mean total cell numbers, mean CD4^+^ cell numbers and mean IFN-γ-producing CD4^+^ T cells in the spleen of control and *Ezh2*-deficient mice 14 days after *Toxoplasm gondii* infection, error bars denote the s.e.m (n* *= 3). Data are representative of three independent experiments ^*^P < 0.05 and ^**^P < 0.01 (unpaired *t*-test).

Our *in vitro* data indicated that *Ezh2-*deficient T cells produced elevated amounts of effector cytokines including IFN-γ and so we sought to investigate the function of *Ezh2*-deficient Th cells in a model that relies on the integrity of Th1 responses. Specifically, we infected mice with *T. gondii*, a parasite known to elicit a strong Th1 response; genetic lesions that cause either impaired or exuberant responses can both result in elevated mortality in this model^28^. *CD4-Cre; Ezh2*^*fl/fl*^ mice together with *Ezh2*^*fl/fl*^ mice were infected intra-peritoneally with an average of 15 parasites. We found that *CD4-Cre; Ezh2*^*fl/fl*^ mice exhibited a significantly increased mortality rate compared with control animals, with the majority of the *Ezh2*-deficient animals dying in the second and third weeks post infection (Fig. 5A). Strikingly though, there was no evidence of an over-exuberant Th1 response. By contrast, when we examined the peritoneal exudate of infected animals, there were more infected cells and monocytes in the *Ezh2-*deficient mice (Fig. 5B), suggesting that the animals were unable to control the *Toxoplasma* infection. Consistent with this, we found that in the spleen and peritoneum of infected *Ezh2-*deficient mice, the percentage and number of CD4^+^ T cells were significantly reduced compared to infected control mice (Fig. 5C). We next analyzed the numbers of IFN-γ and FOXP3 expressing T cells and found that *CD4-Cre; Ezh2*^*fl/fl*^ mice expressed fewer IFN-γ positive Th cells and elevated numbers of FOXP3 expressing Th cells compared with wild type animals, in contrast with our findings *in vitro* (Fig. 5C,D). All these data suggest that the cause of death seen in infected *Ezh2-*deficient mice was not due to an exaggerated inflammatory response but due to a failure to limit parasite burden owing to a suboptimal Th1 response.

In keeping with these data we returned to our transfer colitis model and compared the ability of control and *Ezh2*-deficient naïve T cells to induce transfer colitis. In contrast to mice that received control T cells, mice that received *Ezh2*-deficient T cells maintained their body weight, again suggesting impaired function of effector cells (**Supplementary Fig. 3A**). When we measured the total number of T cells recovered from the animals after four weeks, we found significantly fewer *Ezh2*-deficient cells (**Supplementary Fig. 3B**). Taken together, our data indicate that despite the propensity to produce effector cytokines *in vitro*, *Ezh2*-deficient T cells exhibited inadequate effector responses *in vivo*.

### *Ezh2*-deficient effector T cells rapidly become senescent

**Figure 6:**
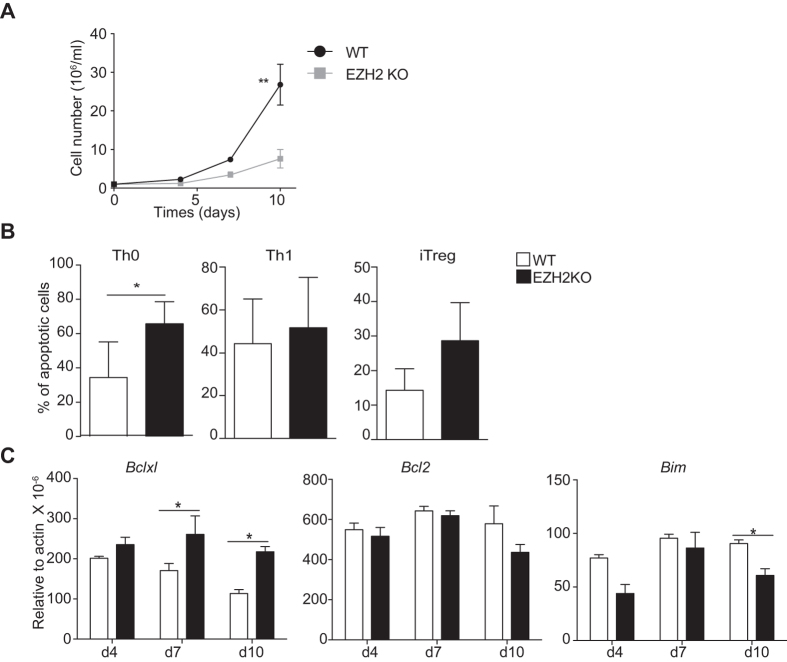
*Ezh2*-deficient T cells fail to expand in long-term culture. Sorted CD4^+^CD44^−^CD62L^+^ cells from control (WT) and *Ezh2*-deficient mice (EZH2KO) were stimulated under Th0, Th1 or iTreg conditions for three days and expanded for a further six days in the presence of IL-2 together with polarising cytokines, (**A**) Total Th1 cell numbers were measured at day 4, 7 and 10, error bars denote s.e.m. ^**^P < 0.01 (unpaired *t*-test) (**B**) Percentage apoptotic cells were determined by flow cytometry measurement of annexin V and propidium iodide after restimulation with anti-CD3 in Th0, Th1 and iTreg polarised cells after 3 days. Histograms indicate the mean percentage of apoptotic cells (±s.e.m) ^*^P < 0.05 (unpaired *t*-test) (n* *= 3) (**C**) *Bcl2, Bclxl, Bim* gene expression was measured by q-PCR at the indicated time points in Th1 cells. Histogram columns denote mean values, error bars denote s.e.m. *P* values were determined using a one way anova, ^*^P < 0.05. Data are reperentative of two independent experiments.

A common feature of *Ezh2*-deficient T cells in the mouse models examined was their failure to expand *in vivo* compared with wild type cells. This contrasted with our findings in short-term culture *in vitro*. To compare the two, we measured cell number after Th1 *in vitro* cell culture over a longer period of time (Fig. 6A). When T cells were allowed to expand in response to exogenous IL-2 we noticed a difference between wild type and *Ezh2*-deficient T cells that became significant after 10 days. It has been suggested that EZH2 antagonizes the expression of pro-apoptotic proteins^14^. We next measured the proportion of annexin V positive *Ezh2*-deficient T cells compared to control T cells grown under Th0, Th1 and iTreg conditions followed by re-stimulation with anti-CD3 and anti-CD28. Although the proportion of annexin V positive cells was consistently higher in the *Ezh2*-deficient cell population this was not significantly so with the exception of cells grown under Th0 conditions (Fig. 6B). To confirm this finding, we also measured a number of regulators of apoptosis including Bcl2, Bcl-XL and Bim (Fig. 6C). Although we could see significant differences in Bcl-XL and Bim expression, these differences were inconsistent with the elevated rates of apoptosis seen in *Ezh2*-deficient cells.

**Figure 7:**
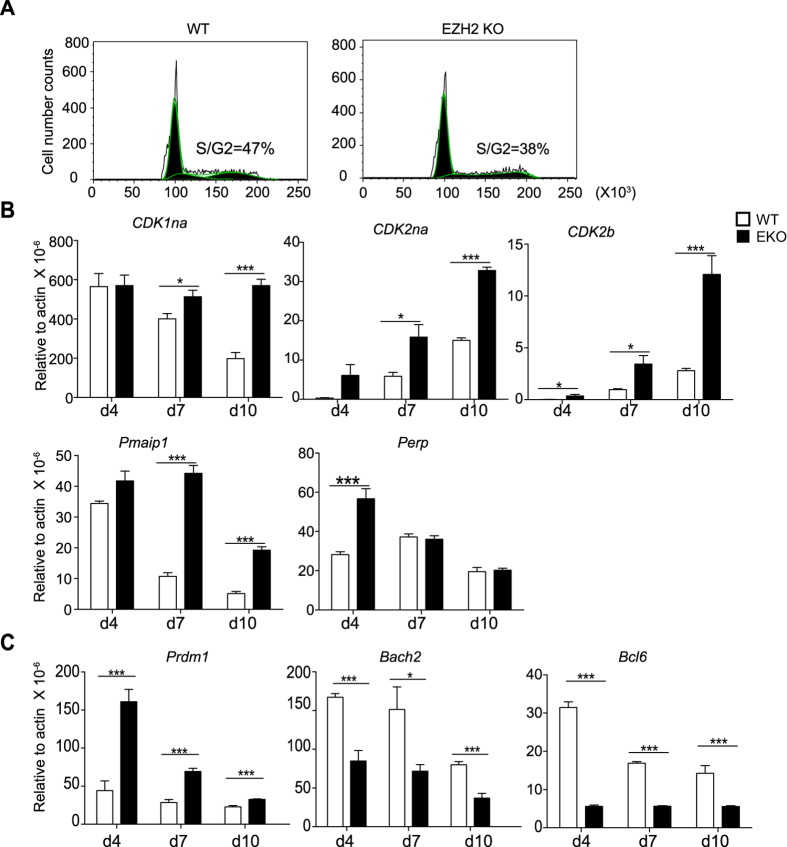
*Ezh2*-deficient T cells have elevated mRNA expression of *Prdm1* and cell cycle inhibitors while they have reduced expression of *Bach2* and *Bcl6*. Sorted CD4^+^CD44^−^CD62L^+^ cells from control (WT) and *Ezh2*-deficient (EZH2KO) mice were stimulated under Th1 for three days and expanded for a further six days in the presence of IL-2. (**A**) On the sixth day of culture, cells were fixed and the live cell population was assessed for DNA quantity using propidium iodide. (**B**) *CDKn1*, *CDKn2a*, *CDKn2b, Pmaip1* and *Perp* gene expression was measured by q-PCR at the indicated time points. (**C**) *Prdm1*, *Bach2* and *Bcl6* gene expression was measured by q-PCR at the indicated time points. Histogram columns denote mean values and error bars denote s.e.m (n* *= 3). *P* values were determined using a one way anova, ^*^P < 0.05, ^***^P < 0.001.

The PRC2 is known to be recruited to its targets in part by Menin^29^. Deficiency of Menin in T cells results in excess cytokine production but is also associated with exaggerated transition to a state of senescence manifested by increased expression of endogenous cell cycle inhibitors, pro-apoptotic factors and increased expression of the transcriptional repressor, Blimp1 (encoded by *Prdm1*)^30^. Therefore we sought to ascertain whether *Ezh2*-deficiency was associated with evidence of premature senescence. We had previously noted that after three days of culture there was no difference in proliferation between control and *Ezh2*-deficient Th cells (Fig. 2). We repeated this experiment under Th1 conditions over six days and noted that at this time point a lower proportion of the *Ezh2*-deficient T cells were in the S/G2 phase of the cell cycle (Fig. 7A). Consistent with these two findings, we found elevated expression of the endogenous cell cycle inhibitors *CDKn1a*, *CDKn2a* and *CDKn2b*, the pro-apoptotic factors *Pmaip1* and *Perp* in *Ezh2*-deficient T cells compared with control cells. Furthermore, with the exception of *Perp*, these differences became more apparent with time (Fig. 7B).

We recently showed that generation of effector cells is constrained by the repressor *Bach2*, which antagonizes the expression of *Prdm1*. We found culture of *Ezh2*-deficient cells resulted in progressive elevation in the expression of Blimp1 and a concomitant loss of Bach2 and Bcl6 expression (Fig. 7C) consistent with Menin-deficient T cells^31^.

## Discussion

Here by specific deletion of *Ezh2* in the T cell compartments we have explored the roles of EZH2 in the regulation of CD4 differentiation: EZH2 suppresses Th1, Th2 and Th17 differentiation but is required for optimal inducible Treg cell generation *in vitro*. By contrast, the effect of *Ezh2* deletion on Treg cells *in vivo* was tissue specific with reduced numbers of FOXP3^+^ T cells in the spleen and lymph nodes, but no difference in Treg numbers in the thymus or lamina propria. In keeping with this, we saw little evidence of the development of spontaneous autoimmune disease, which would be expected with a global defect in Treg cell numbers or function. However, we also documented a role for *Ezh2* in effector cells. Thus under inflammatory conditions such as infection and autoimmunity, EZH2 is critical to maintain T cell homeostasis irrespective of their lineage and function.

Contradictory data pertaining to the role of EZH2 in regulation of CD4 T cell differentiation exist in the literature. Initial work by Koyanagi *et al* suggested that EZH2 promoted the development of Th1 cells via suppression of the alternative Th2 differentiation pathway: EZH2 and H3K27m3 were found to bind the *il4-13* locus to suppress Th2 differentiation during Th1 conditions^20^. Later studies from Jacob and colleagues suggested that EZH2 is recruited to the signature cytokine loci of both Th1 and Th2 cells, namely *Ifng* and *Il4*, but in contrast with prior work, they concluded that the presence of EZH2 enhanced gene transcription at these loci rather than inhibited it^13^,^21^. Despite these mechanistic differences both groups concluded that EZH2 enhanced Th1 differentiation, although both papers were limited by not having access to primary T cells that lacked expression of EZH2.

More recently the role of EZH2 in peripheral T cells has been explored using conditional knock out mice, again with conflicting results: EZH2 has been shown to be a negative regulator of both Th1 and Th2 differentiation leading to accumulation of Th2 memory T cells and increased pathology of allergic asthma^16^. Conversely two groups have shown that despite an increase in the ability of *Ezh2*-deficient to express IFN-γ *in vitro*, EZH2 T cells are unable to cause graft versus host disease or protect against *Leishmania*^14^,^17^. Finally EZH2 has been predicted to play a role in the transcriptional suppressor functions of FOXP3^18^, a master regulator of Treg cells although its role has yet to be determined in *Ezh2*-deficient Treg cells. Consistent with these recent reports^14^,^16^,^17^, we found that in the absence of EZH2, Th1 and Th2 differentiation is significantly enhanced and under neutral conditions, *Ezh2*-deficient cells spontaneously make IFN-γ, suggesting that EZH2 negatively regulates Th1 and Th2 differentiation. In the absence of EZH2, H3K27m3 levels were reduced in both the *Ifng* and *Tbx21* loci under Th1 conditions; the same was observed for *Il4* and *Gata3* under Th2 conditions^16^, arguing a direct role of EZH2 in regulation of those genes. However, there is no H3K27m3 occupancy in the *Foxp3* locus in both naïve and iTreg cells^32^, arguing that EZH2 and H3K27m3 might regulate FOXP3 indirectly. We conclude that EZH2 does not have a direct effect on the *Foxp3* locus, but its reduced expression under iTreg conditions in *Ezh2*-deficient T cells compared with control T cells is due to a failure to restrict the spontaneous expression of IL-4 and IFN-γ in *Ezh2*-deficient T cells stimulated in the presence of anti-CD3/CD28 and TGF-β, a conclusion that is consistent with the recent work of Zhang *et al*^17^. It is of note that when we compared gene expression between control and *Ezh2*-deficient iTreg cells using Gene Set Enrichment Analysis (GSEA) analysis, the family of cytokine genes as a whole was significantly enriched in the *Ezh2*-deficient iTreg cells (data not shown) suggesting that EZH2 plays a role in the restriction of cytokine gene expression. This is consistent with the recent finding that EZH2 and FOXP3 co-localize at multiple genetic loci suggesting that EZH2 plays an important role in the ability of FOXP3 to suppress gene transcription^18^. DuPage *et al.* recently showed that EZH2 is required for maintaining Treg identity and mediating Treg function *in vivo* although *in vitro* the suppressive functions of *Ezh2*-deficient Treg cells appeared to be intact^19^.

Despite the differences in cytokine gene expression and iTreg development that we found *in vitro, Ezh2*-deficient mice did not develop spontaneous autoimmunity. This is in contrast with the work of DuPage and colleagues, where *Ezh2* was deleted in just the Treg cell lineage. We noted that *Ezh2*-deficient mice had a reduced proportion of T cells that were naïve compared with control mice and that this difference increased with the age of the animals. This is a common feature in mice with defects in either numbers or function of Treg cells. Consistent with this finding, there was a reduction in the numbers of FOXP3^+^ T cells in the spleens and mesenteric lymph nodes but not in the colons of *Ezh2*-deficient mice, which may reflect their different origins. In contrast with our findings *in vitro*, there was little evidence of excess cytokine expression from the T cells of *Ezh2*-deficient mice after infection with *toxoplasma* or when they were transferred into *Rag*^*−/−*^ host animals. In both cases we saw evidence of a failure to mount a robust immunological response rather than the expected hypersensitive response, suggesting that the effect of *Ezh2*-deficiency was not limited to impaired Treg cells. The discrepancy could be due to the differential strength of the TCR signaling used in different studies as EZH2 is known to regulate actin polymerization due to a cytoplasmic methytransferase function that leads to a defect in formation of the immunological synapse upon TCR engagement that is associated with a reduction in IL-2 expression^22^. Mutations of *WASP* are associated with actin-related primary immunodeficiency; *WASP*-deficient T cells show a wide range of functional defects including migration, proliferation, and IL-2 production resulting in a substantial decrease in peripheral T cell numbers, and marked reduction of actin polymerization^23^. In this sense, the *Ezh2*-deficient T cells resemble the *WASP*-deficient phenotype. To exclude the involvement of actin polymerization in the regulation of iTreg differentiation *in vitro*, we used *WASP*-deficient CD4 T cells as controls^33^, which also have a defect in actin polymerization. In our hands there was no difference in the proportion of naïve CD4^+^ T cells from control or *WASP*-deficient mice that could differentiate into FOXP3^+^ cells *in vitro* under iTreg conditions, suggesting the reduced iTreg differentiation of *Ezh2*-deficient T cells was not simply due a defect in actin polymerization. Our studies *in vitro* using both *WASP* and *Ezh2*-deficient T cells were performed using anti-CD3 and anti-CD28 antibodies that are unlikely to require the formation of an ordered immunological synapse in order to trigger a robust intra-cellular signaling response. By contrast, our *in vivo* experiments would have required antigen presentation and the formation of an immune synapse.

Failure to form an immune synapse may not be the only reason for the discrepancies that we saw between our *in vivo* and our *in vitro* data. He *et al* have demonstrated that inhibition of EZH2 prevents the development of acute graft versus host disease in mice^14^ and recent work by Zhang *et al* have shown that mice with *Ezh2*-deficient T cells fail to clear *Listeria monocytogenes* infection^17^, consistent with our *in vivo* data. Both groups found that in the absence of EZH2, T cells accumulate pro-apoptotic factors that lead to their failure to proliferate. He *et al* were able to demonstrate that inhibition of Bim expression largely reversed the loss of pathogenicity seen in T cells when EZH2 is blocked^14^ whereas Zhang *et al* identified aberrant activation of caspase 3 and 8 as the principal cause^17^. In our hands, under conditions charecterised by infection and lymphopenia, we found that *Ezh2*-deficient T cells exhibited reduced proliferation and failed to accumulate in the spleen or colon after transfer into *Rag2*^−/−^ mice; and during *Toxoplasma gondii* infection, we found reduced CD4 T cells in the spleen and peritoneal cavity. These results reveal a non-redundant and cell-intrinsic requirement for EZH2 in T cell expansion during an inflammatory response. In short term *in vitro* culture, we saw little difference in cell number between wild type and *Ezh2*-deficient T lymphoblasts but after 10 days the differences in cell numbers were significant. In our hands we did not see an increase in *Bim* expression. However, the percentage of *Ezh2*-deficient apoptotic T cells after *in vitro* culture compared with control T cells was elevated. Consistent with this, we found elevated expression of a number of genes that are associated with senescence together with an elevation in Blimp1 (*Prdm1*) expression and a reduction in *Bach2* expression. Included in our list of cell senescence genes with elevated expression in *Ezh2*-deficient cells list were the pro-apoptotic factors *Perp* and *Pmaip1*^17^,^31^. In many respects, *Ezh2*-deficient T cells behave similarly to *Menin1*-deficient T cells, which seems surprising, as Menin is a member of the trithorax group that opposes the function of the PRC2. However, there is evidence that Menin plays a role in the function of both histone regulatory complexes^29^.

In conclusion, our data highlight many functions of EZH2 in all CD4^+^ T cells not simply the Treg cell lineage and emphasize the pleiotropic nature of this histone modification enzyme. We found that EZH2 plays an important role for Treg cell homeostasis through regulation of expression of many effector cytokines, many of which act to inhibit the expression of FOXP3. Thus, EZH2 is required for optimal iTreg differentiation even in the presence of an excess exogenous IL-2. However, defects in Treg cells are counterbalanced by abnormalities in effector cells. Despite the ability *Ezh2*-deficient T cells to proliferate normally at early time points, their expansion is not sustained. Consequently, robust immune responses do not occur in the absence of EZH2, either in the setting of normal host response or in an model of autommunity. In this setting, EZH2 is required to constrain the program of senescence that occurs in effector cells. Thus EZH2 plays a multifacted role in T cell homeostasis, promoting both regulatory and effector responses.

## Materials and Methods

### Mice and media

*Ezh2*^*fl/fl*^ mice were obtained from the laboratory of Dr. Tarakhovsky as described^22^ and were bred with *CD4-Cre* transgenic mice. These mice were backcrossed with C57BL6 for at least seven generations. Resulting *CD4-Cre*;*Ezh2*^*fl/fl*^ *mice* were further bred to *Foxp3-GFP* reporter mice to generate *Ezh2*^*fl/fl*^*;Foxp3-GFP* mice and *CD4-Cre*;*Ezh2*^*fl/fl*^*;Foxp3-GFP* mice. CD45.1 C57BL6 mice were from Jackson and *Rag*2^−/−^ mice were from NIAID mouse facility (Frederick, MD). All animal studies were performed according to the NIH guidelines for the use and care of live animals and were approved by the Institutional Animal Care and Use Committee of NIAMS. All cell cultures were performed in RPMI with 10% fetal calf serum, 2 mM glutamine, 100 IU/ml penicillin, 0.1 mg/ml streptomycin, Hepes buffer (all Invitrogen, CA) and 2 mM β-mercaptoethanol (Sigma, MO).

### Naïve CD4 T cell isolation and differentiation

CD4^+^ T cells from spleens and lymph nodes of 6- to 8-week-old mice were purified by negative selection and magnetic separation (Miltenyi Biotec, Germany) followed by sorting of naive CD4^+^CD62L^+^CD44^−^ population using FACSAria II (BD, NJ). Cells were activated by plate-bound anti-CD3/CD28 (both 10 μg/ml; eBioscience, CA) in media for 3 days either under neutral conditions or IL-12 (10 ng/ml), anti-IL-4 (10 μg/ml, BD pharmingen) for Th1 differentiation, IL-4 (10 ng/ml), anti-IFN-γ (10 μg/ml, BD pharmingen) for Th2 differentiation, IL-6 (20 ng/ml) plus human TGF-β1 (2.5 ng/ml), anti-IFN-γ (10 μg/ml, BD pharmingen), and anti-IL-4 (10 μg/ml, BD pharmingen) for Th17 differentiation, TGF-β1 (20 ng/ml) and hIL-2 (100 U/ml) for iTreg differentiation unless specified.

### Intracellular staining and flow cytometry

The following antibodies were used: For cell sorting: anti-CD4-PerCPCy5.5, anti-CD62L-APC, anti-CD44-PE and anti-NRP1-APC (all BD, NJ). For intracellular staining: anti-IL-13-PE and anti-IL-17A-PE are from BD (NJ). Anti-IFN-γ-FITC, anti-T-bet-PECy.7, anti-GATA3-PE, anti-RORγt-PE and anti-FOXP3-APC were purchased from eBioscience (CA). In brief, cells were stimulated for 2 hours with PMA and ionomycin with the addition of brefeldin A (GolgiPlug; BD, NJ). Afterwards, cells were fixed with 4% formyl saline, permeabilized with 0.1% saponin buffer and stained with fluorescent antibodies before analyzing on a FACS Verse (BD, NJ). Events were collected and analyzed with Flow Jo software (Tree Star, Ashland, OR).

### Histopathology

Mouse tissues were fixed in 10% formyl-saline followed by embedding in paraffin blocks. Tissue sections were stained in hematoxylin and eosin and reported by a pathologist in a blinded manner.

### Isolation of lamina propria lymphocytes

Large intestines were removed, cleared from mesentery, fat and Peyer’s patches, cut into pieces and washed in HBSS w/o Ca^2+^/Mg^2+^. After incubation in HBSS with EDTA, epithelial layer cells were removed and the remaining tissue was digested with collagenase and DNAse I (both Roche, IN) at 37 °C. Lamina propria lymphocytes were recovered from the supernatant, purified over a 40%, 80% percol gradient and the interface layer washed twice in media before stimulation with phorbol myristate acetate (PMA), ionomycin and brefeldin A.

### *Toxoplasma. gondii* infection and determination of parasite burden

Type II avirulent strain ME49 cysts were obtained from the brains of chronically infected C57BL/6 mice. Cyst preparations were pepsin treated to eliminate potential contamination with host cells and mice were inoculated i.p. with an average of 15 cysts. On day 7 post-infection cytospins were prepared from 1.5 × 10^5^ PEC, fixed and stained. Differential analyses were performed on 200 to 400 cells using an oil immersion (100x) objective.

### Adoptive Transfer Model of Colitis

CD4^+^ T cells were enriched from the spleen and lymph nodes of control (*Ezh2*^fl/fl^) and *Ezh*^−/−^ (*Cd4* Cre; *Ezh2*^fl/fl^) mice with an AutoMACS cell separator (Miltenyi Biotec), stained with PerCP Cy5.5 anti-CD4, FITC anti-CD25, and PE anti-CD45RB (all obtained from BD Biosciences), and naive CD4^+^ CD25^−^ CD45RB^hi^ T cells were purified (>99%) by cell sorting (Moflo,Dako Cytomation or FACSAria, BD Biosciences). CD4^+^GFP^+^CD25^+^NRP1^+^ tTreg cells were purified from *Ezh2*^fl/fl^*Foxp3-GFP* mice or *Cd4* Cre; *Ezh2*^fl/fl^ *Foxp3-GFP.* CD4^+^CD45RB^hi^CD25^−^ naive T cells (4 × 10^5^) from control mice and *Ezh2*-deficient mice were injected intravenously (i.v.) into age- and sex-matched *Rag2*^−/−^ mice and intestinal inflammation was monitored. Alternatively, *Rag2*^−/−^ mice were coinjected with 4 × 10^5^ naive T cells and 1 × 10^5^ CD4^+^CD45RB^lo^CD25^+^ Treg cells from control mice and *Ezh2*-deficient mice. Mice were sacrificed at 7 weeks when significant weight loss occurred in the control groups. Gut cells were isolated as described previously by intracellular staining. Sections of the proximal, mid-, and distal colon were fixed in buffered 10% formalin and stained with hematoxylin and eosin (H&E) (Histoserv).

### Statistics

GraphPad Prism 6.0 was used for statistical analysis.

## Additional Information

**How to cite this article**: Yang, X.-P. *et al.* EZH2 is crucial for both differentiation of regulatory T cells and T effector cell expansion. *Sci. Rep.* **5**, 10643; doi: 10.1038/srep10643 (2015).

## Supplementary Material

Supplementary Information
