# Supplementary material for: EZH2 is crucial for both differentiation of regulatory T cells and T effector cell expansion
Source: Sci Rep. 2015 Jun 19;5:10643. doi: 10.1038/srep10643 (PMC4473539; doi:10.1038/srep10643)
Supplement: Supplementary Information [file srep10643-s1.pdf]

**Supplementary information for EZH2 is crucial for both differentiation of regulatory T cells and T effector cell expansion**

Xiang-Ping Yang[1, 2, 7*], Kan Jiang[2, 7], Kiyoshi Hirahara[2], Golnaz Vahedi[2], Behdad Afzali [2,3], Giuseppe Sciume[2], Michael Bonelli[2], Hong-Wei Sun[4], Dragana Jankovic[5], Yuka Kanno[2], Vittorio Sartorelli[6], John J O'Shea[2], Arian Laurence[2*]

[1] Department of Immunology, School of Basic Medicine, Tongji Medical College, Huazhong University of Science and Technology, Wuhan, China, 430030 [2]Lymphocyte Cell Biology Section, Molecular Immunology and Inflammation Branch, National Institutes of Arthritis, and Musculoskeletal and Skin Diseases National Institutes of Health, Bethesda, MD 20892, USA, [3]MRC Centre for Transplantation, King's College London, UK, [4]Biodata Mining and Discovery Section, National Institute of Arthritis and Musculoskeletal and Skin Diseases, National Institutes of Health, Bethesda, MD 20892-1930, USA, [5] Immunobiology Section, Laboratory of Parasitic Diseases, National Institute of Allergy and Infectious Diseases, [6]Laboratory of Muscle Stem Cells and Gene Regulation, National Institute of Arthritis and Musculoskeletal and Skin Diseases, National Institutes of Health, Bethesda, MD 20892-1930, USA, [7]These authors contributed equally.

* Corresponding authors: Email: yangxp@hust.edu.cn, laurencea@mail.nih.gov

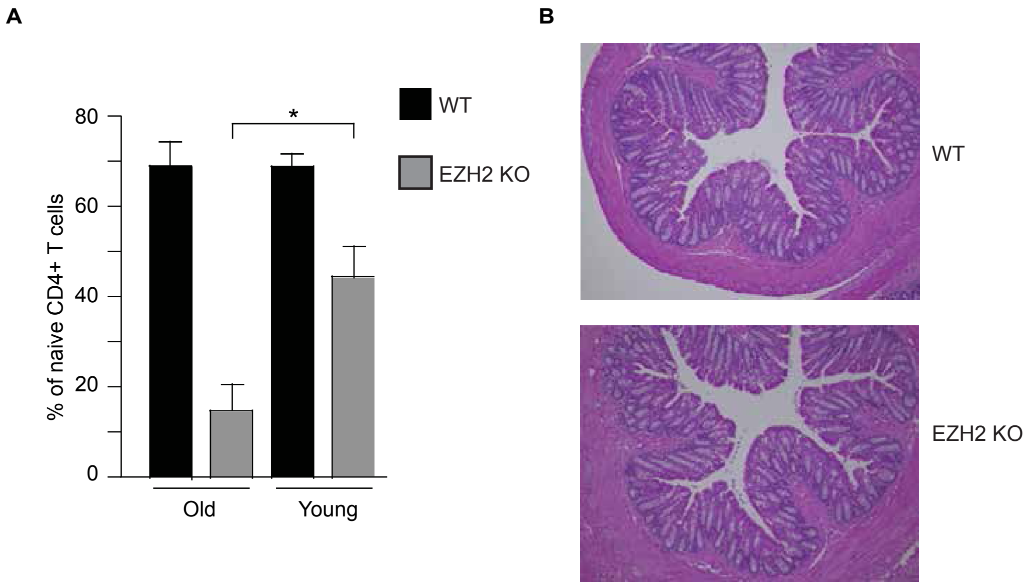


**Ezh2-deficient mice have fewer naïve T cells in their periphery with age.**

The spleen and mesenteric lymph nodes were isolated from Ezh2-deficient mice at age 6 weeks and 26 weeks, followed by red blood cell lysis. The isolated lymphocytes were stained for cell surface marks of CD4, CD62L, and CD44. (A) Cumulative histogram showing percentage of $CD4^{+}CD44^{low}CD62L^{high}$ and $CD4^{+}CD44^{high}CD62L^{low}$ in the spleen of Ezh2-deficient mice at age 6 weeks (young) or 26 weeks (old). *P < 0.05 and **P < 0.005 (unpaired t-test). (B) The histopathology staining of the colons of 6 month old control mice and Ezh2-deficient mice. Mouse tissues were fixed in 10% formyl-saline followed by embedding in paraffin blocks and tissue sections were stained in hematoxylin and eosin.

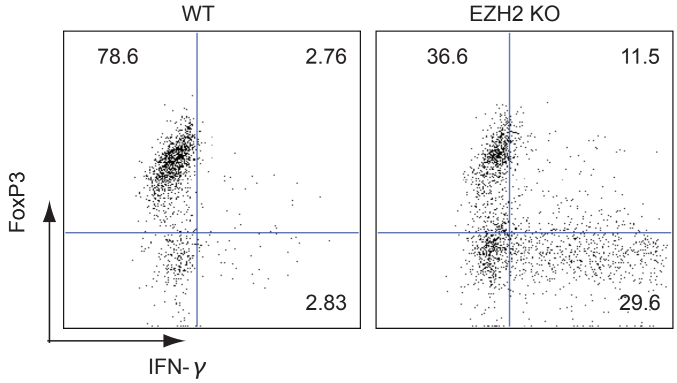


**Destablization of nTreg cells in the absence of EZH2**

Sorted splenic $CD4^{+}CD25^{+}nrp^{+}$ cells from WT mice or EZH2-deficient mice were stimulated with anti-CD3/CD28 (10 μg/ml) and IL-12 (10 ng/ml) for 3 days, expressions of FoxP3 and IFN-γ were determined by flow cytometry.

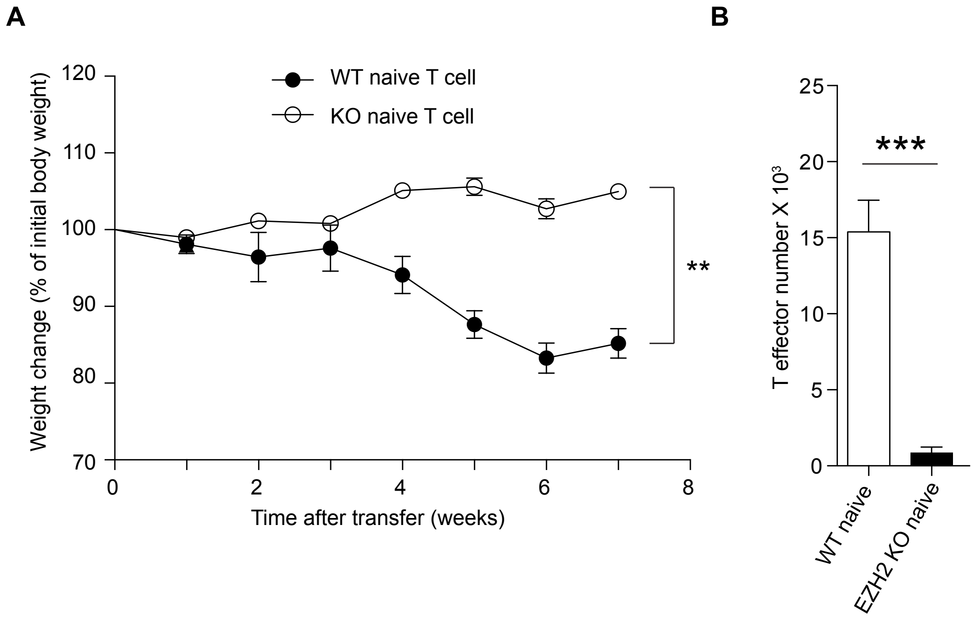


**Ezh2-deficient T cells failed to induce colitis.**

Sorted $4x10^5$ $CD4^+CD45RB^{hi}CD25^-$ cells from CD45.1 control mice or sorted $4x10^5$ $CD4^+CD45RB^{hi}CD25^-$ cells from Ezh2-deficeint mice were injected intravenously into 6-8 weeks Rag2-/- mice alone (n=5). Mice were monitored for evidence of colitis. (A) Weight loss (percentage of initial weight at day 0) was calculated for each mouse over 8 weeks. Data show mean (±SEM) weight changes for each group (n = 3–5 mice) (**P < 0.01). (B) Total lymphocyte cells were recovered from the lamina propria. **P < 0.01 (unpaired t-test).
